# A 24‐Year‐Old Male Adult With a Severe Intellectual Developmental Disorder and Intractable Challenging Behaviors: A Case Report

**DOI:** 10.1155/crps/5963356

**Published:** 2026-05-30

**Authors:** Edouard Guez, Nicolas Turin, Filippia Doulou, Yoann Beigheldrut, Pia El Kossaifi, Frank Ferrari, Michèle Levy-Soussan, David Cohen, Marianna Giannitelli

**Affiliations:** ^1^ Department of Child and Adolescent Psychiatry, Pitie-Salpetriere Hospital, AP-HP, Sorbonne University, Paris, France, sorbonne-universites.fr; ^2^ Pierre Louis Institute of Epidemiology and Public Health (iPLesp), INSERM UMR-S1136, Mental Health and Addictions (ESSMA) Team, Social Epidemiology, Sorbonne University, Paris, France, sorbonne-universites.fr; ^3^ Ethical Support Unit, Pitie-Salpetriere Hospital, AP-HP, Sorbonne University, Paris, France, sorbonne-universites.fr; ^4^ Institute for Intelligent Systems and Robotics (ISIR), CNRS UMR 7222, Sorbonne University, Paris, France, sorbonne-universites.fr

**Keywords:** autism, case report, multidisciplinary care, restraint

## Abstract

**Background:**

This case highlights the clinical complexity of caring for individuals with autism spectrum disorder (ASD) and severe intellectual developmental disorder (IDD), particularly in the context of prolonged mechanical restraint, psychotropic polypharmacy, and life‐threatening somatic complications. It illustrates how a multidisciplinary and individualized neurobehavioral approach can lead to major clinical improvement despite an initially poor prognosis and limitations regarding intensive care.

**Case Presentation:**

Nathan is a 24‐year‐old man of Slavic origin with ASD and severe IDD who had been subjected to continuous mechanical restraint for 2 years and was receiving five concomitant antipsychotic medications. He presented with severe behavioral dysregulation in a context of chronic anxiety, marked communication limitations, recurrent aspiration pneumonia related to gastrostomy feeding, and adverse effects associated with antipsychotic treatment. He was referred to a specialized neurobehavioral unit, where a multidisciplinary team—including psychiatry, intensive care, palliative care, and ethics specialists—agreed on a conservative management plan. Antipsychotic treatment was streamlined to clozapine, fluoxetine was later introduced, and care combined nutritional rehabilitation, communication‐focused support, and individualized psychoeducational, occupational, and psychomotor interventions in a highly structured environment. Oral feeding was gradually reintroduced, ultimately allowing gastrostomy removal, and restrictive measures were progressively discontinued. Overtime, his behavioral symptoms, emotional regulation, physical health, mobility, and overall quality of life improved markedly.

**Conclusions:**

This case underscores the importance of integrated multidisciplinary care in complex ASD/IDD presentations involving severe behavioral and somatic complications. Psychopharmacological simplification, environmental and communication adaptations, and individualized rehabilitation strategies were central to the improvement in both somatic outcomes and behavioral functioning. It also highlights the need to recognize prolonged mechanical restraint as a harmful and unsustainable management strategy in individuals with severe neurodevelopmental disorders.

## 1. Introduction

In most countries, the use of physical restraint in hospital settings for psychiatric patients is governed by a strict legal framework [1]. In France, restraint may only be used when a patient poses an imminent risk to themselves or others and when other de‐escalation strategies, including medication, have failed. Its use must also be clearly justified and documented. The ethical issues surrounding physical restraint have been widely discussed, and experts consistently recommend verbal de‐escalation and environmental modification as first‐line strategies. Physical restraint is considered a measure of last resort [2]. Similarly, pharmacological treatment should be selected by carefully balancing potential benefits against adverse effects, such as oversedation. In patients with autism, invasive intensive care procedures may themselves require restraint or sedation because of limited understanding and reduced tolerance of such interventions. Major ethical concerns related to restraint therefore also arise in resuscitation and intensive care settings [3–5]. The intensity of care should primarily depend on the patient’s vital prognosis. Disease burden, organ dysfunction, and the number of medical devices have been identified as key predictors of severity in adults and older adults admitted to intensive care [6]. In this context, severe neurodevelopmental disorders, together with associated behavioral and emotional symptoms, raise complex questions about what level of resuscitative care can, should, or should not be provided.

The neurobehavioral unit within the Department of Child and Adolescent Psychiatry at Pitie‐Salpetriere Hospital in Paris provides medical and psychoeducational care for patients with autism spectrum disorder (ASD), intellectual developmental disorder (IDD), and severe challenging behaviors. Care includes psychiatric and somatic management, as well as psychological, psychomotor, and educational support, with multiple behavioral de‐escalation strategies aimed at minimizing the use of restraint [7]. We report the case of a young adult who was admitted after years of escalating interventions for challenging behaviors, including antipsychotic polypharmacy, prolonged physical restraint, and gastrostomy following recurrent aspiration pneumonia. This case raised several intertwined clinical and ethical questions: (i) concern among both the family and psychiatric teams that intensive care might be withheld solely because of the patient’s severe neurodevelopmental disorder; (ii) difficulty in recognizing the specific challenges associated with invasive intensive care in a patient with ASD and severe IDD; (iii) a sense of helplessness regarding the assessment and management of critical somatic complications within a psychiatric setting; (iv) difficulty in defining the most appropriate level of care across teams with different clinical cultures.

## 2. Case Presentation

**Figure 1:**
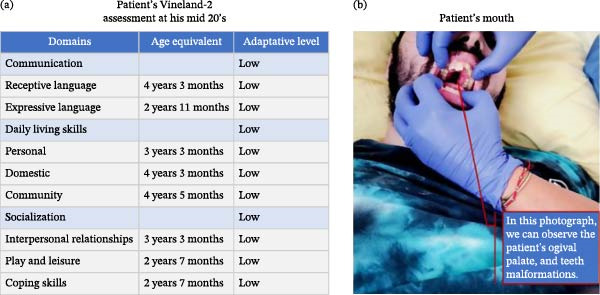
(a) Patient’s developmental and adaptive assessment (Vineland‐2) and (b) patient’s mouth.

Nathan, a 24‐year‐old man of Slavic origin with ASD and severe IDD (Figure 1a), was subjected to continuous physical restraint from August 2021 to September 2023. He was also treated with five antipsychotic medications that were introduced sequentially and ultimately maintained concomitantly. In addition, a gastrostomy tube was placed in late 2021 because of severe swallowing difficulties, thought to be mainly related to adverse effects of antipsychotic treatment, including anticholinergic, extrapyramidal, and sedative effects, as well as central motor dysfunction. Prolonged supine positioning further worsened the dysphagia.

He was born 70 days preterm in Eastern Europe, weighing 1700 g, and had an inguinoscrotal hernia, right convergent strabismus, and gastroesophageal reflux at birth. His developmental history was marked by global psychomotor delay, and ASD and IDD were diagnosed at 3 years of age. There was no history of developmental regression, but challenging behaviors had been noted since his adoption at 2 years of age. He was able to understand simple routine verbal instructions. Between 4 and 5 years of age, he developed separation anxiety, sleep disturbances, anorexia, and tantrums involving moderate self‐injurious and aggressive behaviors. Nathan lived in a residential care setting from 8 to 14 years of age. He also had a history of dysphasia and orofacial abnormalities (Figure 1b).

From a psychological and communicative perspective, Nathan showed a chronic anxiety‐prone profile with marked fluctuations between periods of relative calm and periods of severe nervousness and behavioral dysregulation. He displayed a strong and often poorly regulated need for exclusive attention from caregivers, while remaining only minimally engaged with peers. His oral language was present but highly ritualized, with repetitive questions and stereotyped utterances mainly centered on meals, activities, and daily routines. He was nevertheless able to initiate interactions verbally, understand simple instructions in context, and produce short multi‐word sentences, although his answers were often limited to “yes” or “no.” Emotionally, he appeared to have major difficulty identifying and regulating affect, and often expressed frustration, sadness, or anger through repetitive questioning, shouting, or aggression. He also presented numerous stereotypies and restricted interests, while still showing interest in activities such as listening to music, walking, and coloring. Formal psychological assessment during hospitalization supported this clinical profile, with the Vineland‐II (Figure 1a) documenting globally low adaptive functioning, including a receptive communication age‐equivalent of 4 years 3 months and an expressive communication age‐equivalent below 3 years (2 years 11 months). ComVoor nevertheless showed preserved learning abilities and supported the use of bidimensional pictorial supports and a visual schedule, while direct behavioral observation suggested that his challenging behaviors were primarily driven by social attention seeking, frustration, and marked anticipatory anxiety.

At 9 years of age, an etiological work‐up, including testing for fragile X syndrome, karyotyping, metabolic investigations, and chromosomal microarray analysis, revealed no abnormalities. He later experienced a seizure and was started on valproic acid at a dose of 1 g/day. During adolescence, antipsychotic treatment was progressively escalated, both in dose and in the number of medications prescribed, to manage his challenging behaviors. In early adulthood, he was admitted to a residential care facility, where he exhibited marked challenging behaviors, including self‐injurious behaviors and aggression. These behaviors appeared to be driven largely by anxiety and ineffective communication strategies. He had previously enjoyed activities such as coloring, listening to music, and walking. Overtime, his dysphagia worsened and was accompanied by marked weight loss.

### 2.1. Clinical Findings

After gastrostomy placement in late 2021, Nathan experienced six episodes of aspiration pneumonia secondary to vomiting during enteral feeding between December 2021 and March 2023. These events required multiple admissions to pulmonology and gastroenterology departments at his local hospital. In January 2022, Nathan removed his gastrostomy tube himself, and a winged gastrostomy tube was reinserted under sedation. In June 2023, he was referred to gastroenterology for severe fecal impaction associated with iron deficiency and hyperkalemia. During the COVID‐19 period, his parents’ visits were interrupted, a change that likely worsened his challenging behaviors, including self‐injurious behaviors. These behaviors led to a head injury with concussion in August 2021 and to infection of a posttraumatic hematoma of the right elbow in September 2021. As a result, several recreational activities were discontinued, and he was transferred to a psychiatric facility, where soft restraints were initiated because of severe aggression toward others. After gastrostomy placement, what had initially been introduced as a crisis‐management measure progressively became chronic, and by January 2023, the restraint system had been reinforced with more restrictive mechanical restraints to prevent him from removing the gastrostomy tube. In retrospect, this prolonged use of restraint represented a major departure from recommended practice and was likely to have contributed to physical deconditioning, reduced access to meaningful activities, and worsening behavioral dysregulation. A short summary of the main clinical events is provided in Table 1.

**Table 1:** Clinical timeline of the main events from prolonged restraint to multidisciplinary improvement at the neurobehavioral unit.

Date/period	Main clinical event/intervention	Clinical outcome/relevance
Early childhood	Prematurity, early developmental delay; ASD and IDD diagnosed at 3 years	Baseline neurodevelopmental vulnerability; challenging behaviors noted from early childhood
2019	Seizure episode; valproic acid introduced	Neurological comorbidity treated
August–September 2021	Severe self‐injurious behavior with concussion and infected posttraumatic elbow hematoma; soft restraints initiated	Recreational activities reduced; transfer to psychiatric care
Late 2021–January 2022	Worsening dysphagia; gastrostomy placed; tube later removed by the patient and repositioned under sedation	Enteral feeding increased need for restrictive measures
December 2021–March 2023	Six aspiration pneumonia episodes related to vomiting during enteral feeding	Repeated medical admissions and life‐threatening somatic burden
January–June 2023	Mechanical restraints reinforced; severe fecal impaction, iron deficiency, and hyperkalemia documented	Escalating restrictive care and cumulative somatic complications
July 2023	Admission to the neurobehavioral unit under restraint and antipsychotic polypharmacy; vomiting, desaturation, and aspiration pneumonia shortly after admission	Multidisciplinary care planning; conservative monitoring strategy within the neurobehavioral unit
August–September 2023	Antipsychotic regimen simplified; clozapine titrated; long‐acting antipsychotics discontinued; oral feeding resumed; gastrostomy removed	Progressive discontinuation of restraint and improvement in somatic status
October–November 2023 onward	Fluoxetine introduced; structured psychoeducational, psychomotor, and communication‐focused care continued; respiratory episode managed	Improved anxiety, behavior, mobility, participation, and quality of life; future high‐dependency monitoring could be considered if needed

*Note:* It summarizes the main clinical events, somatic complications, treatment changes, and care decisions from the onset of prolonged restraint and gastrostomy‐related complications to clinical improvement under neurobehavioral multidisciplinary care.

### 2.2. Timeline and Management in the Neurobehavioral Unit

**Figure 2:**
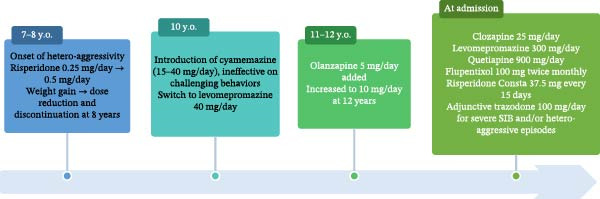
Course of antipsychotic treatment from childhood onward.

Nathan was admitted to our unit in July 2023 for assessment and management of a life‐threatening condition related to recurrent somatic complications and ongoing restrictive measures. At admission, his treatment regimen included clozapine (25 mg/day), levomepromazine (300 mg/day), quetiapine (900 mg/day, reduced to 800 mg/day on admission), valproic acid (1000 mg/day), long‐acting flupentixol (100 mg twice monthly), and long‐acting risperidone (37.5 mg every 15 days). In addition, trazodone 100 mg/day was authorized as needed for severe episodes of agitation and aggression. Figure 2 summarizes the course of Nathan’s antipsychotic treatment from childhood onward.

Shortly after admission, Nathan experienced two episodes of vomiting and desaturated to 88% on room air. Chest imaging suggested right‐sided pulmonary involvement, which we hypothesized was related to impaired airway clearance, possibly exacerbated by positional factors. He also developed aspiration pneumonia, prompting renewed discussion about the appropriate level of care.

### 2.3. Diagnostic Assessment and Multidisciplinary Management

A multidisciplinary consultation was held at Pitie‐Salpetriere Hospital involving the Palliative Care, Medical Ethics, Infectious and Tropical Diseases, Medical Intensive Care, and Child Psychiatry teams. The aim was to define an appropriate care plan for Nathan and to facilitate discussion between psychiatrists and intensive care physicians regarding their respective concerns. Three main issues were discussed: (i) whether invasive intensive care would be appropriate in the event of respiratory deterioration; (ii) how to balance somatic safety, quality of life, and the harmful effects of prolonged restraint; (iii) how to establish a coherent treatment plan that could be discussed with the family in the context of a life‐threatening clinical situation. The child psychiatry team recommended reducing antipsychotic treatment, implementing close follow‐up to address behavioral difficulties and progressively discontinue restraints, and removing the gastrostomy tube in order to improve Nathan’s quality of life. The infectious diseases and intensive care teams initially considered that invasive ICU management would be poorly tolerated and of uncertain benefit in a context of prolonged mechanical restraint, major iatrogenic burden, and severely impaired quality of life. At that stage, because Nathan showed no major signs of acute respiratory distress, they favored close clinical monitoring and reevaluation rather than immediate escalation to invasive intensive care. The multidisciplinary discussion was therefore essential in clarifying the goals of care, sharing responsibility across teams, and organizing a treatment strategy that could be reevaluated according to his clinical evolution.

### 2.4. Therapeutic Intervention

It was, therefore, decided that both somatic and neurobehavioral care would be provided within the neurobehavioral unit, with the following anticipatory measures: (i) the initial agreed plan was to avoid immediate escalation to invasive ICU care and to prioritize close clinical monitoring within the unit, with reevaluation according to Nathan’s clinical evolution; (ii) pulmonologists recommended close clinical monitoring within the unit; (iii) from a nutritional perspective, oral feeding was gradually reintroduced using thickened water and a pureed diet. Following clinical improvement over the ensuing weeks, the gastrostomy tube was removed in September 2023.

### 2.5. Follow‐Up and Outcomes

**Figure 3:**
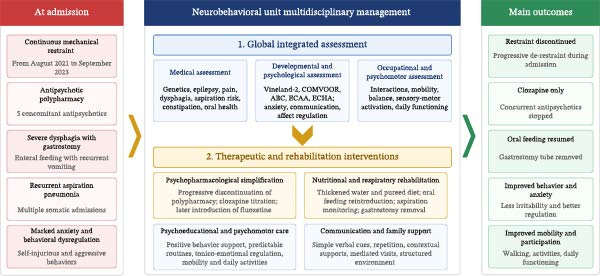
Multidisciplinary intervention pathway and main outcomes at the neurobehavioral unit. *Note*: Left‐to‐right flow diagram summarizing baseline clinical burden at admission, multidisciplinary assessment, major therapeutic and rehabilitation interventions at the neurobehavioral unit, and the main outcomes. The figure links the initial situation (continuous restraint, antipsychotic polypharmacy, dysphagia with gastrostomy, recurrent aspiration pneumonia, and severe behavioral dysregulation) to integrated assessment, psychopharmacological simplification, nutritional and respiratory rehabilitation, psychoeducational and psychomotor care, communication and family support, and the main clinical outcomes (restraint discontinuation, clozapine monotherapy, oral feeding resumption with gastrostomy removal, and improvement in behavior, anxiety, mobility, and participation).

The multidisciplinary intervention pathway and the main outcomes observed at the neurobehavioral unit are summarized in Figure 3. Behavioral management relied on a combination of psychopharmacological adjustment and individualized psychoeducational, occupational, psychomotor, and communication‐focused interventions. Antipsychotic treatment was simplified to clozapine, which was titrated from 25 to 250 mg/day in accordance with international recommendations, while concomitant psychotropic medications and long‐acting injectable antipsychotics were progressively discontinued. Fluoxetine was subsequently introduced because of suspected depressive symptoms and was associated with reduced anxiety and irritability. Combined with nutritional and respiratory rehabilitation, restoration of meaningful activities, supportive relational interventions, and a highly structured environment, these changes contributed to a marked improvement in challenging behaviors, emotional regulation, mobility, and daily participation. Because of Nathan’s severe intellectual and communicative limitations, including an expressive communication developmental level below 3 years on the Vineland‐II, no standardized PTSD assessment was performed, and no formal PTSD diagnosis was made. The team nevertheless adopted a trauma‐informed approach and explicitly considered the possibility of trauma‐related manifestations linked to prolonged restraint and repeated coercive somatic care. Clinical attention was, therefore, paid to indirect indicators such as marked anxiety, hyperarousal, behavioral worsening in stressful or restrictive contexts, intolerance of change, and progressive improvement after reduction of coercion, restoration of predictable routines, reintroduction of meaningful activities, and supportive relational interventions. Communication was supported through simple verbal instructions, repetition, contextual cues, PECS–based pictorial supports, and a highly structured care environment. At discharge, a new multidisciplinary meeting was held. In light of Nathan’s clinical progress, the intensive care team reconsidered their initial position: in the event of future respiratory distress, admission to a continuous care or high‐dependency unit for optimized ventilation and close monitoring could be considered.

## 3. Discussion

This case highlights the challenges involved in managing severe challenging behaviors in adults with ASD and severe IDD, as well as the potential benefits of specialized multidisciplinary care. Interventions in adults with complex neurodevelopmental disorders often have limited effects, with particularly modest improvements reported for self‐injurious behaviors and stereotypies, and somewhat greater benefits for emotional regulation [8]. In this context, our case is of particular interest because it raises two closely related issues: the prolonged use of restraint and difficult decisions regarding intensive care.

In France, the General Controller of Places of Deprivation and Liberty (https://www.cglpl.fr/) states that seclusion and restraint should be used only in exceptional circumstances, when a crisis cannot be resolved by other means [1]. Because a crisis is, by definition, time‐limited, such measures should be used only for the shortest period necessary to restore safety or implement an alternative strategy. The same report also emphasizes that isolation and restraint in the patient’s own room should be avoided because of the risk of normalization and insufficient traceability [1]. These recommendations are important for understanding the context of Nathan’s management. He remained under continuous restraint for 2 years in his residential care facility before these measures were progressively discontinued following improvement in his somatic condition and behavioral functioning in the neurobehavioral unit. In this respect, Nathan’s prolonged continuous mechanical restraint for nearly 2 years cannot be regarded as acceptable routine care. What had initially been introduced as an emergency measure progressively became chronic, despite the principle that restraint should remain exceptional, brief, and limited to the shortest period necessary [1, 2]. The subsequent improvement observed after psychotropic simplification, psychoeducational rehabilitation, restoration of activities, and progressive discontinuation of restraint suggests that long‐term mechanical restraint had become part of the problem rather than part of the solution. We, therefore, consider this prolonged restrictive management to have been profoundly inappropriate, potentially harmful, and likely traumatic.

Several studies have shown that neurobehavioral units dedicated to the management of challenging behaviors in autistic individuals can lead to meaningful improvements in quality of life [7, 9, 10]. These units generally rely on a multidisciplinary model of care [7]. Understanding challenging behavior requires a functional and multidimensional assessment, while crisis management typically combines educational and behavioral approaches [10] with medical interventions when necessary [11, 12]. In Nathan’s case, management required simultaneous consideration of developmental, adaptive, environmental, somatic, behavioral, and emotional factors. Identifying the mechanisms contributing to his behavioral dysregulation was essential and helped guide care over several months. In addition, trauma‐related manifestations must be considered in autistic individuals with IDD, particularly after prolonged exposure to restraint and repeated coercive care, even when formal PTSD assessment is difficult because of severe communication limitations. In such situations, a trauma‐informed approach may rely less on standardized verbal reporting than on indirect behavioral indicators, environmental predictability, reduction of coercion, and support for communication. This case also highlights the need for anxiety and trauma assessment tools adapted to autistic individuals with severe IDD and limited verbal abilities. Recent hospital‐based studies [13, 14] support individualized autism‐specific care pathways combining environmental modification, communication accommodations, and structured behavioral strategies. In perioperative settings, individualized plans based on communication style, triggers, and prior experiences, together with quiet rooms, reduced transitions, limited personnel, dimmed lights, child life support, and tailored anxiolysis, have been shown to be feasible and well accepted [13]. In psychiatric inpatient settings, autism‐specific care pathways emphasizing predictability, visual supports, total communication, activity, and reward‐based strategies have also been positively perceived by staff, with visual communication aids and reward strategies identified as particularly helpful [14]. The strategies used at the neurobehavioral unit were consistent with this framework.

Regarding intensive care in individuals with ASD and IDD, the literature remains limited [15]. First, individuals with IDD appear more likely to receive cardiopulmonary resuscitation at the end of life, possibly because do‐not‐resuscitate orders are used less frequently in this population [16]. Questions related to restraint also extend to resuscitation and intensive care settings [3–5], although they take a different form from those encountered in psychiatric practice. In principle, prognosis in intensive care primarily depends on the severity of organ failure, although ethical questions may still influence decisions regarding ICU admission [6]. In Nathan’s case, given the prolonged use of restraint in a psychiatric setting, a conservative multidisciplinary approach was chosen despite recurrent life‐threatening episodes of aspiration pneumonia. Consultation with the palliative care unit and the medical ethics team was crucial in establishing a shared therapeutic approach [12]. Each team recognized the constraints faced by the others: psychiatrists sought support for the management of severe somatic complications, whereas intensivists emphasized the likely burden and uncertain benefit of invasive measures in the context of prolonged restraint and severely impaired quality of life, initially favoring a conservative and closely monitored approach. To our knowledge, only one comparable case has been reported in the literature: a 22‐year‐old inpatient with ASD and IDD who developed respiratory failure and required admission to the intensive care unit [15]. In that case, the patient improved through integrated interventions focused on environmental adaptations and communication strategies. Similarly, Nathan’s case suggests that close collaboration between medical and psychiatric teams can be beneficial. In light of his subsequent clinical improvement, intensivists who had initially ruled out ICU admission later agreed that future admission to a continuous care setting could be considered if needed.

Our experience with Nathan, as well as with other patients requiring complex somatic care, underscores the need for further research to better guide intensive care decision‐making in individuals with ASD and IDD.

## 4. Conclusion

We report the case of a patient with ASD, severe IDD, and life‐threatening somatic complications whose management had come to rely on prolonged mechanical restraint. His condition improved through close collaboration between psychiatric and somatic teams, combining psychopharmacological simplification, psychoeducational and behavioral interventions, nutritional management, and respiratory monitoring. This case highlights the need to recognize prolonged mechanical restraint as a harmful and unsustainable management strategy in individuals with severe neurodevelopmental disorders.

**Nomenclature**
ASD: Autism spectrum disorder
ICU: Intensive care unit
IDD: Intellectual developmental disorder
mg/d: Milligrams per day
m.: Months
PECS: Picture exchange communication system
y.: Years
y.o.: Years old.

## Author Contributions

Edouard Guez was involved in the patient’s clinical management and drafted the manuscript. Nicolas Turin contributed to the patient’s management and to manuscript preparation. Filippia Doulou conducted the psychological assessment and contributed to the psychological aspects of the manuscript. Yoann Beigheldrut conducted the main educational assessment and contributed to the corresponding section of the manuscript. Pia El Kossaifi conducted the occupational assessment, contributed to the related section, and translated the manuscript from French into English. Frank Ferrari and Michèle Levy‐Soussan contributed to the ethical aspects of the patient’s management. David Cohen critically revised the manuscript. Marianna Giannitelli was responsible for the patient’s care and supervised the manuscript.

## Funding

The authors received no specific funding for this work.

## Disclosure

This perspective is based on written exchanges with Nathan’s mother during and after his hospitalization. She remained closely involved throughout his care, participated in meetings with the team, shared videos and previous medical reports to help clinicians better understand her son’s baseline functioning, and provided consent for key procedures and for this anonymized publication. Her messages reflected both concern about Nathan’s somatic fragility, particularly during episodes of aspiration and pneumonia, and hope that his treatment burden and daily difficulties could be reduced. She also contributed concretely to his rehabilitation by proposing practical supports, such as providing an exercise bike for the unit. Overall, these exchanges reflected sustained caregiver involvement and strong concern for her son’s quality of life.

## Ethics Statement

This case report was conducted in accordance with the principles of the Declaration of Helsinki.

## Consent

Written informed consent was obtained from the patient’s legal guardians for publication of this case report and the accompanying images. A copy of the written consent is available for review by the Editor‐in‐Chief of this journal.

## Conflicts of Interest

The authors declare no conflicts of interest.

## Data Availability

Data sharing is not applicable to this article because no datasets were generated or analyzed during the current study.
